# Interorganisational collaboration to improve accessibility of diagnostic evaluations for children

**DOI:** 10.1093/eurpub/ckac131.014

**Published:** 2022-10-25

**Authors:** E Cloet, A Jansen, M Leys

**Affiliations:** 1 Public Health, Vrije Universiteit Brussel, Jette, Belgium; 2 Paediatric Neurology, Universitair Ziekenhuis Brussel, Jette, Belgium; 3 Paediatric Neurology, Universitair Ziekenhuis Antwerpen, Antwerpen, Belgium

## Abstract

**Background:**

Children with a suspected developmental disability need early diagnostic evaluations and support, to maximize developmental opportunities. Accessibility to diagnostic settings in Flanders, Belgium, is poor, with waiting periods up to two years. Interorganisational coordination of activities using a public health and needs of the population perspective is needed to strengthen the system. This study aims to evaluate current practices and opportunities for interorganisational collaboration of organisations active in the field of diagnostics.

**Methods:**

It concerns a qualitative, policy-support research project. 6 homogeneous focus groups were organised for the 6 types of organisations subsidized by the government to perform diagnostic evaluations of children with a (suspected) developmental disorders. Data were thematically analysed and categorized in a process of researcher and data-triangulation. A member check validation was done.

**Results:**

59 persons participated. We classified the hampering and facilitating factors for collaboration Current interorganisational collaboration is mostly limited to referral. Organisational differences in vision and goals, working processes and quality requirements, regulations and financial support criteria, a problem of accessibility and communication problems are hampering factors. Knowledge of mutual expertise and working processes, personal contacts and open communication are facilitating factors.

**Conclusions:**

A population public health based organisation of the field of multidisciplinary diagnostic evaluations for children with a developmental disorder, with interorganisational collaboration and coordination of activities in mandated networks, sharing experience and knowledge, would increase the accessibility for all children and strenghten the health system. The implementation of interorganisational networks would benefit from a functional and institutional analysis of organisations.

**Key messages:**

• Coordination of activities and interorganisational collaboration in mandated networks, could potentially improve the accessibility of diagnostic evaluations for children with a developmental disorder.

• Implementation of interorganisational networks would benefit from a functional and institutional analysis of organisations.

